# The role of the refractory period in diapause length determination in a freshwater crustacean

**DOI:** 10.1038/s41598-019-48389-6

**Published:** 2019-08-15

**Authors:** Mirosław Ślusarczyk, Wojciech Chlebicki, Joanna Pijanowska, Jacek Radzikowski

**Affiliations:** Department of Hydrobiology, Faculty of Biology, University of Warsaw, Center of Biological and Chemical Research, Żwirki i Wigury 101, 02-089 Warszawa, Poland

**Keywords:** Evolutionary ecology, Evolutionary developmental biology, Zoology, Ecophysiology, Freshwater ecology

## Abstract

We investigate here the mechanism of allochronic resumption of development by the dormant forms in organisms inhabiting temporary habitats. The cohorts of resting eggs of a short living freshwater crustacean *Daphnia magna* collected in two temporary waters at two occasions (spring and autumn) were exposed after different storage periods (0–16 weeks) spent either in wet or dry conditions to a given set of hatching stimuli announcing appearance of favourable conditions. Freshly formed resting eggs did not hatch or hatched occasionally. The resting eggs formed in autumn hatched more eagerly than the spring ones when exposed to favourable conditions after wet storage. The hatching proportion increased linearly up to 68–82% in autumn resting eggs while to 33–44% in the spring ones over 16 weeks of storage that might have covered several generations of the active forms. Dry storage of the resting eggs reduced their hatching proportion considerably. We suspect that the length variation of a refractory period (initial phase of developmental arrest when resting forms remain insensitive to hatching stimuli) followed by a reactivation period may constitute the simplest two-step physiological mechanism allowing staggering revival of the dormant forms in subsequent generations that maximise chances for survival in unpredictably changing habitats.

## Introduction

Most living creatures on Earth that occupy temporarily deteriorating habitats suspend development during inhospitable periods. This mechanism is usually referred to as diapause in animals and physiological dormancy in plants. Due to the restrained functionality of the organisms and the lower physiological demands on them during these periods, the diapause (called also resting) stage may rely on less effective but more robust metabolic pathways allowing them to retain functional integrity over a broader range of environmental conditions compared to active forms^1^. To be effective, the diapause should be initiated at the appropriate time. The onset should occur prior to the deterioration of environmental conditions and the process should terminate when conditions improve again. A too-early onset may increase the costs of diapause to an unmanageable degree, while a delay in onset would expose individuals to the deteriorating conditions. Moreover, too-early diapause termination would expose individuals to unfavourable conditions while late diapause termination would increase diapause costs. For these reasons timing of diapause may remain under strong selective pressure.

While the mechanism of diapause induction attracts more scientific attention^2–4^ the mechanism of diapause termination remains understudied except for organisms important in agriculture (crops, weeds and pests). In the present study, we seek to achieve a better understanding of the mechanism behind diapause termination. This process seems to be more simple than its initiation, given that dormant forms do not need to anticipate environmental changes when changing their developmental modes (as in case of diapause induction) but simply track them and resume development when conditions improve. Yet the stochasticity of future environmental conditions during the growing season may make the decision of diapause termination challenging as well. Organisms may employ various precautions against making a reckless decision to resume development; one of these may be a bet-hedging mode (sensu Seger & Brockman^5^) of the diapause termination. Such a mechanism would diversify the length of diapause between dormant forms, thus facilitating genome protection against occasional failures in survival or reproduction of post-diapause forms during the growing seasons.

Cohen^6^ was likely the first theoretician to explore the optimum length of dormancy in annual organisms. His initial study was further developed by Bulmer^7^ and Ellner^8^. They concluded that developmental arrest in highly predictable (e.g. seasonal) locations should last as short as possible and terminate immediately following the improvement of environmental conditions in their habitat. Some dormant forms might extend their developmental arrest over a growing season or seasons if the environmental conditions do not allow for survival or reproduction in some growing periods. The higher the risk of unsuccessful reproduction during the growing season, the higher the incidence of postponed development of dormant forms until the next seasons. In their simulation studies, Menu *et al*.^9^ and Ślusarczyk *et al*.^10^ considered longer diapause periods than the earlier models and revealed that short living univoltine organisms inhabiting unpredictably changing habitats may use a diversified length of developmental arrest to promote long term survival of their genetic lines. Both studies claimed the existence of an evolutionary stable strategy (ESS), resulting in the formation of diversified offspring which postponed their development from 0 to a few generations. The greater the fluctuation in environmental conditions and/or the lower the mortality of dormant forms, the more variable should be the length of developmental arrest^10^.

In the present study, we focused on the putative mechanism of diapause termination. The simplest mechanism of diapause termination might be an inspection of environmental conditions by the dormant form and the subsequent resumption of development with the return of favourable environmental conditions^1^. Such a mechanism may be supported by a conditional process determining the length of a refractory period^1^ also called a maintenance phase in animals^11^ or physiological dormancy in plants^12^, i.e. a temporal phase in which the dormant forms remain insensitive to stimuli initiating their development. Such a refractory period may require specific termination conditions to prevent the premature development of dormant forms e.g. temporal heating, freezing, inundation, smoke or exposure to fire, or even nothing more than temporal sojourn in given conditions (for review see^1,12,13^). Across the cohort of dormant forms in a population, the threshold levels of the conditional parameter that terminates the refractory phase may be similar or different, depending on level of uncertainty of the environmental conditions. In the first case, all dormant forms would resume development at the same time as they are exposed to favourable hatching/germinating stimuli. Different threshold level of the conditional parameter would diversify the length of the diapause period under favourable conditions and would result in an asynchronous resumption of development named germ banking^13^. We suspect that the length diversification of the refractory period may be the simplest mechanism allowing gradual revival of the dormant forms in subsequent generations and diversifying the risk of faulty choice of activity in unpredictably changing habitats.

Variable length of refractory period lasting 0–3 generations have been reported in insects^14–16^) and annual plants^17^. In terms of generation time, longer refractory phase have been reported in organisms with shorter lifespan, e.g. in planktonic fairy shrimps^18,19^ or copepods^20^.

We hypothesized that some other short living organisms - planktonic cladocerans of the genus *Daphnia* inhabiting temporary waters - may use a refractory period to diversify the minimum length of diapause of their resting eggs. Existing data may support this view indicating a significant effect of storage period on hatching proportion of the resting eggs in *Daphnia*^21,22^. We aimed to verify this view by analysing the length of the refractory periods of resting eggs formed on two occasions (in spring and autumn) by *Daphnia magna* originating from two temporary urban ponds in a temperate climate. We will place these results within the context of the predictions made in our recent simulation study^10^.

## Materials and Methods

### Object of the study

*Daphnia* are filter-feeding multivoltine organisms living in open water of freshwater lentic habitats. Most *Daphnia* do not live for more than a few months and several generations may succeed in a growing season^23^. Under favourable conditions, *Daphnia* mitotically form multiple viviparous eggs which hatch into females or, less commonly, males; both are genetically identical to their mothers. Sex in this group is determined hormonally, not genetically^24^. When conditions deteriorate, some females intensify the production of males. Other females form meiotic eggs, which, after being fertilised, halt their development and remain in stasis, enclosed in a protective chitinous shell called an ephippium. During the next molt, the ephippium containing the dormant eggs is released into the water column or onto the surface; there where they remain in stasis for an indefinite time until conditions favourable for development arise. The ephippia released into the water column commonly sink to the bottom, while those oviposited at the surface are more likely to be dispersed to other habitats by various vectors such as animals, wind or water^25^. Some species of *Daphnia* may asexually produce both immediately hatching (subitaneous) and resting eggs^26^. The species used in the current study, *D*. *magna*, inhabits temporary small, fish-free bodies of water and is most likely to form dormant eggs in a sexual way.

### Ephippia collection and processing

We collected both ephippial and nonephippial females of *D*. *magna* with a plankton net during the two annual periods of intense ephippia formation in late May and late October 2014, in two unnamed urban ponds located in recreational parks in the centre of Warsaw, Poland: Park Na Książęcem (GIS: 52.2305N, 21.0284E) and Park Ujazdowski (GIS: 52.2212N, 21.0260E), called hereafter PK and PU, respectively. Both ponds are relatively small (0.3 ha - PK, 0.7 ha - PU), shallow (max. depth 0.8 m - PK and 1.5 m - PU) and manmade. Both ponds have solid bottoms made of concrete (PK) or stones (PU). PU contains water throughout the year except for in the early spring when it is drained for a few days for bottom cleaning and sediment removal. Sometimes, water is not drained throughout the whole year in PU. PK seems to offer less predictable conditions then in PU. It is typically drained for cleaning in November and remains dry for 4–6 months till spring when it is refilled with tap water. Due to more erratic management program than that of PU, it may occasionally dry out in hot summers. Unfortunately, we do not have regular data on hydroperiods in the two ponds. *Daphnia* reappear regularly in each pond after water drainage and refill, most likely due to resurrection from local bank of resting eggs that remains after imperfect sediment cleaning or due to their immigration from neighbourhood location aided by rich community of waterfowls. The ponds are uninhabited by fish although occasionally citizens release their pet fish into them. Such incidental fish stocks are unable to increase over the long term due to the temporal drainage of the ponds, and the released fish appear to pose no significant threat to *Daphnia* unless water is not drained. *Daphnia* in these ponds are, however, exposed to other threats, both biotic (invertebrate predation, parasitism, food deterioration) and abiotic (water chemistry changes, drying or freezing) ones in rather unpredictable manner.

We transferred the collected *Daphnia* in their original pond water to separate aquaria in the lab where they were kept for 36 h at a low temperature (16 °C) and a long photoperiod (16 L:8D), to allow them to shed their ephippia. The incubation time of 36 h is too short to allow *Daphnia* to form and release new ephippial eggs in the lab at that temperature. Thus, all ephippia and resting eggs used in the further tests were induced and formed in the field and later released in the lab, what allowed us to control their age. The ephippia released in the aquaria were collected. Most of collected ephippia that were opened (about 40 at each occasion from each pond) contained two healthy looking resting eggs. The remaining intact ephippia were split haphazardly into two groups: those from the first group (named hereafter “dry”) were dried on a filter paper for 24 h in a dark climate room at 16 °C and then transferred dry into Eppendorf vials, with 10 randomly collected ephippia in each vial. Ephippia from the other group (named hereafter “wet”) were transferred from the aquaria into the Eppendorf vials filled with 1 ml of filtered (50μm) pond water, with 10 randomly assembled ephippia per vial. Then, prior to further treatments, these wet ephippia were kept together with the drying ones for 24 h in a dark climate room at 16 °C. Ephippia from both dry and wet groups from two locations (PK/PU) and two seasons (spring/autumn) were split further randomly into 9 groups with different storage time treatments. Altogether we tested about 14 400 ephippial eggs (2 ponds x 2 seasons x 2 storage quality treatments (dry/wet) x 9 storage time treatments x 10 replications (Eppendorf vials) x 10 ephippia in each vial x 2 ephippial eggs in each ephippium).

The Eppendorf vials used in the first storage time treatment, containing “wet” ephippia or rehydrated “dry” ephippia and named hereafter “0” were transferred to a common incubation chamber and exposed to the same hatching stimuli (15 ± 0.5 °C, high intensity of fluorescent light (≈600 lux) at spring photoperiod (16 L:8D)). We found these incubation conditions to be effective hatching stimuli in a former study^27^. The Eppendorf vials with dried ephippia were filled with 1 ml of the filtered (50μm) pond water prior to incubation (the same source of water has been used as for ephippia kept wet). The remaining of the tested ephippia (both dry and wet) were moved into a dark climate room (5 °C) for 1, 2, 3, 4, 6, 8, 12 or 16 weeks prior to incubation.

The Eppendorf vials were inspected every two days during incubation. The hatched individuals were counted and removed from the vials as they appeared and the water was refilled. Ephippia from each group were incubated for one month until hatching had nearly ceased. The hatching proportion was calculated as the number of hatched individuals out of maximum potential number of ephippial eggs (20) in each eppendorf vial. We could therefore slightly underestimate this way the hatching proportion of the ephippial eggs once not all ephippia could contain two viable eggs prior incubation. The further procedure inclined however this bias as rather minor. Following the incubation period, all ephippia were opened in 5 of 10 Eppendorf vials in each treatment and the number of remaining eggs was determined. The eggs were qualified as viable or non-viable based upon visual inspection. The ephippial eggs, considered as viable, had intact chorion and uniform greenish colour content, while non-viable eggs had yellowish, inconsistently coloured content or ruptured chorion.

The hatching dynamics were compared between treatments using a GLM generalized linear model with defined binomial error distribution and logit link function (with R software Ver. 3.4.2^28^). We defined in the model as qualitative variables the pond of origin of the tested ephippia (PK vs. PU), the season of ephippia formation (spring vs. autumn) as well as the storage method (wet vs. dry), while the storage time in the dark cool room was defined as a continuous covariate. The maximal model contained all main effects and all interactions. In the present study we compared features of ephippia originating from limited number of independent samples. For instance neither seasons nor ponds of origin of the ephippia were replicated in quantities that would allow to draw more general conclusion about their deterministic role. Therefore, some of the independent variables (pond of ephippia origin, season of ephippia formation) were used as discriminative features of analysed biological material rather than explanatory variables in our analysis. We need to be cautious in interpreting our results.

## Results

**Figure 1:**
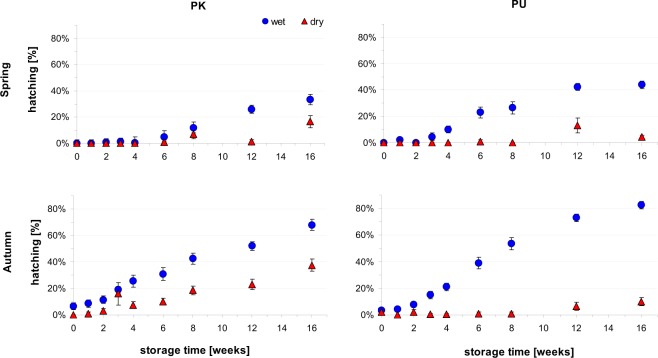
The hatching success (mean ± 1SE) of ephippial eggs of *Daphnia magna* collected in spring and autumn in two temporary ponds (PK and PU) after different storage periods either in dry or wet conditions in a cool dark room.

In both ponds, the ephippia collected in the spring did not hatch under favourable incubation conditions without having been previously stored in the cool dark room (Fig. 1).

Short-term wet storage (of up to 4 weeks in the case of PK and up to 2 weeks in case of PU) did not lead to any increase in the hatching success above null. Longer storage time progressively increased the hatching success by up to 33% for PK and up to 44% for PU after 16 weeks of wet storage (Fig. 1). In contrast, some ephippial eggs formed in autumn and stored wet, were ready to hatch without temporal storage a few days after egg formation (6.5% for PK and 3.5% for PU). The proportion of resting eggs that hatched during the incubation period increased linearly with storage time. The maximum hatching rates (68% for PK and 82% for PU) were achieved following the longest applied wet storage time i.e. 16 weeks. Dry storage in the cool dark room decreased hatching success of the ephippial eggs significantly when compared to the wet storage condition in the relevant time treatments (Fig. 1, Table 1).

**Table 1:** The GLM table (indicating significance of factors and their interactions in a maximal model) of the hatching proportion of *D*. *magna* ephippial eggs formed in the two seasons (spring vs. autumn) in two ponds (PK vs. PU) and stored for different periods of time (sttime 0–16 weeks) in dry/wet storage conditions (stcond) in a cool and dark room prior to incubation.

	Estimate	Std. Error	z value	Pr(>|z|)
**Coefficients:**				
(Intercept)	−3.205330	0.145180	−22.078	<2e-16***
pond	−1.931506	0.371771	−5.195	2.04e-07***
stcond	1.062833	0.175728	6.048	1.46e-09***
season	−3.062907	0.492210	−6.223	4.88e-10***
sttime	0.174038	0.013711	12.694	<2e-16***
pond:stcond	1.454825	0.400750	3.630	0.000283***
pond:season	2.024266	0.774574	2.613	0.008965**
stcond:season	0.509360	0.555226	0.917	0.358937
pond:sttime	0.009123	0.031501	0.290	0.772104
stcond:sttime	0.019176	0.017844	1.075	0.282526
season:sttime	0.112706	0.037083	3.039	0.002371**
pond:stcond:season	−0.087755	0.835571	−0.105	0.916357
pond:stcond:sttime	0.097832	0.036413	2.687	0.007215**
pond:season:sttime	−0.045684	0.059507	−0.768	0.442666
stcond:season:sttime	−0.036103	0.043241	−0.835	0.403764
pond:stcond:season:sttime	−0.112640	0.066464	−1.695	0.090124

glm (formula = y ~ pond * stcond * season * sttime, family = binomial).

Signif. codes: 0 ‘***’ 0.001 ‘**’ 0.01 ‘*’ 0.05 ‘·’ 0.1 ‘’ 1.

Null deviance: 4842.2 on 719 degrees of freedom.

Residual deviance: 1242.0 on 704 degrees of freedom.

AIC: 2287.4.

All four explanatory variables (pond, season, storage time and storage conditions) and some interactions between them revealed significant effect on hatching proportions of ephippial eggs of *D*. *magna* (Table 1). Significant interaction between storage conditions and pond of origin of the tested ephippia may indicate various distance between mean hatching intensity of dried and wet ephippial eggs originating from the two ponds (with higher difference between dried ephippia). Significant interaction between ponds and seasons may indicate various distance of overall mean of hatching frequency of ephippial eggs (when lumped wet and dry eggs together) originating from the two ponds between seasons. Significant three way interaction between the origin of tested ephippia, storage conditions and seasons may be more difficult to interpret and might reflect different effect on hatching proportions of storage conditions between resting eggs originating from the two ponds and different seasons (e.g. lower hatching proportion of dry ephippial eggs but not the wet ones between PU and PK in autumn yet not in spring).

Visual inspection of the unhatched ephippial eggs after incubation indicated that most of them remained viable after incubation periods (Fig. 1, Supplementary Data).

## Discussion

The results of our study: (1) confirmed the existence of a refractory period occurring during the suspended development of *D*. *magna* ephippial eggs originating from temporary waters, (2) challenged existing reports of shorter refractory periods of dried than wet ephippial. eggs of *D*. *magna*, and (3) documented considerable variability in the length of the refractory period within cohorts of the resting eggs from given location and season. Among the various possible components comprising this variability (i.e. genetic, developmental, environmental factors), one may be an inherited strategy allowing for the diapause length diversification. Such an adaptation has been claimed to represent an optimum^6,7^ or evolutionary stable strategy^8–10^ serving to facilitate the long-term persistence of genetic lines in unpredictably changing habitats. While most theoreticians have argued that a relatively short diapause period, lasting one or a few generations, might be adaptive^6,9^, our recent simulation study indicated the adaptive value of longer dormancy periods when environmental conditions are highly variable or when mortality of dormant forms is low^10^. The diversified length of bouts of suspended development with long refractory periods more effectively facilitated the long-term persistence of genetic lines of virtual organisms in variable habitats than did the strategy of short term dormancy.

In both ponds, a few ephippial eggs formed in autumn were ready to hatch a few days after shedding, while about 20% remained in the refractory period for 16 weeks following wet storage (longer refractory periods were not tested in our study due to time limitation). The proportion of hatching ephippial eggs increased steadily as the storage time passed (Fig. 1), indicating that a constant proportion of ephippial eggs completed the refractory period per time unit when being stored in invariable conditions. All of these data may provide support for the conclusions of our recent simulation study^10^, where, in unpredictably changing habitats, the most fit genetic lineages of virtual organisms formed diversified types of offspring which remained in diapause for different periods of time, either in a proportion that was constant (in highly variable habitats or at low mortality of dormant forms) or decreasing with time (in less variable habitats or at considerable mortality of the dormant forms with time). In that study, the diversified bet-hedging strategy of developmental arrest over generations offered a competitive advantage and promoted the long-term persistence of organisms with those traits in the varying habitats^10^. A slightly different scenarios was reported in earlier studies on *Daphnia* as well as other planktonic crustaceans^20–22^. For instance Moreira dos Sanatos and Persoone^22^ tested hatching dynamics of ephippial eggs of *D*. *magna* collected in an English pond during period of intense ephippia formation in spring. Some of them hatched readily without storage period (56%) while their storage in the darkness at 4 °C affected significantly their hatching fraction that reached 25% after 1 month, 37% after 2 months and 60% after 3 months of storage. Unlike in our present study, the high proportion of ephippia were ready to hatch without delay in spring. Unfortunately, neither characteristics of the native pond nor the way ephippia were collected in the field are presented in their paper. Moreira dos Santos and Persoone^22^ also reported that warmer storage in dark conditions (8 months at 4 °C vs 20 °C) decreased hatching fraction of ephippial eggs of *D*. *magna* from 55% to 30% respectively. Similar trend has been reported by De Meester and De Jagger^29^ a few years earlier. Drying of ephippial eggs either increased^30^ or decreased^21^ hatching rates of the resting eggs of *D*. *magna* in some earlier studies.

The maximum hatching rates of resting eggs of freshwater cladocerans inhabiting permanent habitats is typically lower compared to ones inhabiting temporary waters^27,31^ and rarely exceeds 40% (but see Weider *et al*.^32^ for exceptionally high hatching rate of the lacustrine *Daphnia*). In some other crustaceans (branchiopods *Branchinella longirostris* and *Paralimnadia badia*) from temporary waters, lower hatchlings proportion could be observed under common garden conditions within dormant forms originating from habitats with more predictable seasonal changes and longer hydroperiod compared to ones from less predictable and more variable habitats^33^.

A female of *D*. *magna* may form at most 2 ephippial eggs within a single clutch; this is hardly sufficient to provide diversity in the lengths of their periods of developmental arrest. A clonal lineage of *Daphnia* may likely form an array of ephippial eggs with each having a different refractory period length. This needs verification, however. In the current study, these observed differences ranged from a few days to a few months, during which time, short-living *Daphnia* may form several generations. In favourable conditions, with non-limiting resources and optimum water temperature, *D*. *magna* generation time may be as short as a single week (personal observations). In the natural habitat, this short developmental period may be considerably extended due to suboptimal environmental conditions, e.g. low water temperature or scarcity of resources.

As we have suggested, the diversified length of diapause in *Daphnia* appears to be adaptive in habitats which are exposed to strong unpredictable fluctuations of environmental conditions^10^. Both of the urban ponds tested in our study are man-made bodies of water which are drained once a year at regular intervals (for PK this occurs in the winter and for PU - in the spring). Yet their small size (both in terms of area and depth) and strong anthropopressure, qualify them as highly variable habitats exposed to considerable and unpredictable changes in abiotic (temperature, salinity, water level) and biotic (food quality and quantity, predation pressure, intra- and interspecific competition, parasitism) conditions which are known to cause high fluctuations in population size of *D*. *magna* during a growing season^34^.

We found a significant difference in hatching dynamics of ephippial eggs according to storage time between ponds and seasons as well as between storage conditions. Surprisingly, the minimum length of the refractory period was shorter in *D*. *magna* ephippial eggs formed during the autumn than during the spring in each of the two ponds. While we expected longer refractory period in autumn than in spring to prevent premature hatching in the middle of winter, some wet ephippial eggs formed in autumn were ready to hatch without a storage prerequisite (6.5% - PK and 3.5% - PU), while the resting eggs formed in spring required at least a few weeks of storage to initiate hatching (6 weeks in case of PK and 3 weeks in case of PU). The readiness of the autumn-formed resting eggs to respond to the hatching stimuli after only a few days of storage does not necessary imply that they are reactivated early in the field. Under natural conditions, the second hypothetical prerequisite of successful reactivation – the presence of hatching stimuli (e.g. inundation, light exposition and elevated temperature) may not appear until a few months after autumn, in the spring. The bet-hedging pattern of reactivation of ephippial eggs formed in autumn is rather surprising, especially in case of *Daphnia* from PK pond. Environmental conditions in PU pond may be variable in winter. The winter period with ice cover may vary considerably between years with occasional winters without ice cover and mild environmental conditions favourable for growth and reproduction of *Daphnia*. Reasons of bet-hedging pattern of ephippial eggs formed in autumn in PK are more puzzling since the pond is typically drained in autumn and hardly ever offers favourable condition in winter. Frequent displacement of *Daphnia* ephippia between urban ponds by aquatic birds may likely mix populations between ponds and slow down unique adaptations to local environmental conditions.

On the other hand, we see good reasons to postpone for at least few weeks reactivation of the resting eggs newly formed in the late spring. In the early spring exephippial *Daphnia* typically encounter favourable conditions for growth and reproduction with abundant algae food level of good quality. In that time population of *Daphnia* may rapidly increase numerically due to high reproductive effort, short generation time and parthenogenesis. In turn, within a few weeks, the population size of *Daphnia* may exceed the carrying capacity of the habitat when not being top-down controlled by fish, leading to starvation and further population decline. A few weeks later, environmental conditions may improve again coupled with an increase in algal biomass, as the decimated population of *Daphnia* has been unable to control the algae^34^. While the long term prospects for development during the growing season are uncertain the bet-hedging diapausing strategy may be selected for, due to spring overcrowding the nearest opportunity for reproduction following ephippial formation is set a few weeks later; for this reason most if not all resting eggs formed in spring may enter a refractory period lasting at least a few weeks. This hypothesis needs verification, however. We are currently unable to exclude alternative explanations for the observed variability, e.g. some uncontrolled external factors may have affected the results of our study in the spring vs. autumn seasons (hatching experiments on spring and autumn resting eggs were conducted in different time due to inevitable experimental constrains and thus, some uncontrolled experimental conditions may be responsible for observed seasonal difference); or perhaps genetic differences in the population of *Daphnia* exist within and between seasons.

In this study, all of the ephippial eggs were stored prior to incubation in cool conditions (4–5 °C). In small bodies of water similar conditions may occur in the autumn but not in the spring when the water temperature is higher. According to Moreira dos Santos and Persoone^22^, storage of *Daphnia* eggs at a higher temperature should extend the refractory period and diminish observed hatching success after a given storage period. Dry storage in cool dark condition and following incubation under wet conditions reduced the hatching success of ephippial eggs compared to the resting eggs stored and incubated under wet conditions in our study. This supports some earlier findings^35^ while challenges some others^30^. For unclear reason the refractory phase was shorter for ephippia stored in the dry form originating from PK then PU. According to theoretical expectations^6,7,10^ higher variability and unpredictability of environmental conditions in PK pond should rather select for lower and not higher hatching rates compared to PU, as being observed. Most of the ephippial eggs which did not hatch during the incubation procedure appeared to be viable following extended storage period under dry or wet conditions (Fig. 1 supplementary data), indicating that the time travellers maintained themselves in the refractory state and waited for future occasions for development.

While we have not investigated the physiological mechanism responsible for the different length of the refractory periods in the resting eggs of *Daphnia*, we suspect some two-step mechanism of their reactivation. The first step - maternally controlled variable length of the refractory period, might diversify revival of the progeny over generations, whereas the second step, controlled by the embryo – would scan for favourable period of their reactivation. Ephippial mothers could deposit in the resting eggs or in their external structures (ephippial wall) some chemical compound inhibiting their response to hatching stimuli, which decomposes over time (e.g. light-absorbing dark pigmentation). The mothers could manage the length of the refractory period of their resting eggs by providing them with different amounts of the inhibitory substance. We do not know the chemical nature of the alleged substance, but we speculate that the rate of its decomposition might decrease as the temperature increases, what may explain the results of Moreira dos Santos & Persoone^22^. As indicated by Pancella & Stross^36^ sodium hypochlorite treatment may terminate refractory period and likely deactivate the putative substance in *Daphnia*. The chemical composition of this hypothetical substance and the physiological mechanism itself, require further investigation, however.

## Supplementary information

Dataset1

## Data Availability

The datasets analyzed during the current study are available from the corresponding author on request.
