# Supplementary material for: The role of the refractory period in diapause length determination in a freshwater crustacean
Source: Sci Rep. 2019 Aug 15;9:11905. doi: 10.1038/s41598-019-48389-6 (PMC6695402; doi:10.1038/s41598-019-48389-6)
Supplement: Supplementary file 1 — Dataset1 [file 41598_2019_48389_MOESM1_ESM.doc]

**The role of the refractory period in diapause length determination in a freshwater crustacean**

Mirosław Ślusarczyk, Wojciech Chlebicki, Joanna Pijanowska & Jacek Radzikowski

**Supplementary data, Fig 1.**

Proportion of viable and nonviable eggs classified upon visual scoring after the final hatching trial.
